# Supplementary material for: Comparative Population Genomics of Relictual Caribbean Island Gossypium hirsutum
Source: Mol Ecol. 2026 Jan 14;35(2):e70239. doi: 10.1111/mec.70239 (PMC12801185; doi:10.1111/mec.70239)
Supplement: Supplementary file 1 — Figure S1: Population genetic structure analysis used for outgroup selection and interpretation of diversity within G. barbadense , using (A) PCA, (B) Cross‐entropy values vs. the best ancestral populations (K), and (C) neighbour‐joining analysis, LEA structure results (with K = 4) and cpDNA type. In (C), tree tips are labelled with sample ID. The nine G. barbadense individuals selected are highlighted with a black dash bix in the phylogeny and genetic structure plot. Figure S2: Heatmap plot of genetic relatedness (PI_HAT > 0.3) between all 86 samples from Mound Key (MK), Guadeloupe (GD; n = 21), and Puerto Rico (PR_CR, PR_Ph with n = 5, PR_Hwy325). Richer colour intensity indicates higher degree of relatedness. Figure S3: Cross‐entropy values for the structure plot. Each dot represents an average cross‐entropy value for each number of ancestral populations (K) across 10 replicates; standard errors are represented by a vertical bar across each dot. Figure S4: TreeMix model selection with migration edges ranging from 0 to 8, with each model was replicated 10 times. The best model (m = 7) was selected based on Δm. Figure S5: (A) Maximum parsimony phylogenetic tree for all 158 samples using the whole plastome and excluding one of the two copies of the large inverted repeat. Each tree tip is labelled by a shape and an individual ID that represents their genetic group, which was assigned using genome‐wide SNPs (see Figure 1A and Figure S1A). Clades are coloured based on assigned groups. (B) Haplotype network using the same plastome alignment, and the colours represent their cpDNA main types. Figure S6: Maximum likelihood trees for (A) 110 loci dataset and (B) whole plastome. Nodes with bootstrap value > 95 black dots, and nodes between 85 and 95 black dots. Figure S7: (A) Averaged proportions of ROH across all individuals in each population/group. (B) Correlations between the number of ROH and the proportion of the genome occupied by ROH. [file MEC-35-e70239-s002.zip › mec70239-sup-0006-FigureS5@FigS5_n158_cotton_wholeplastome_revised.pdf]

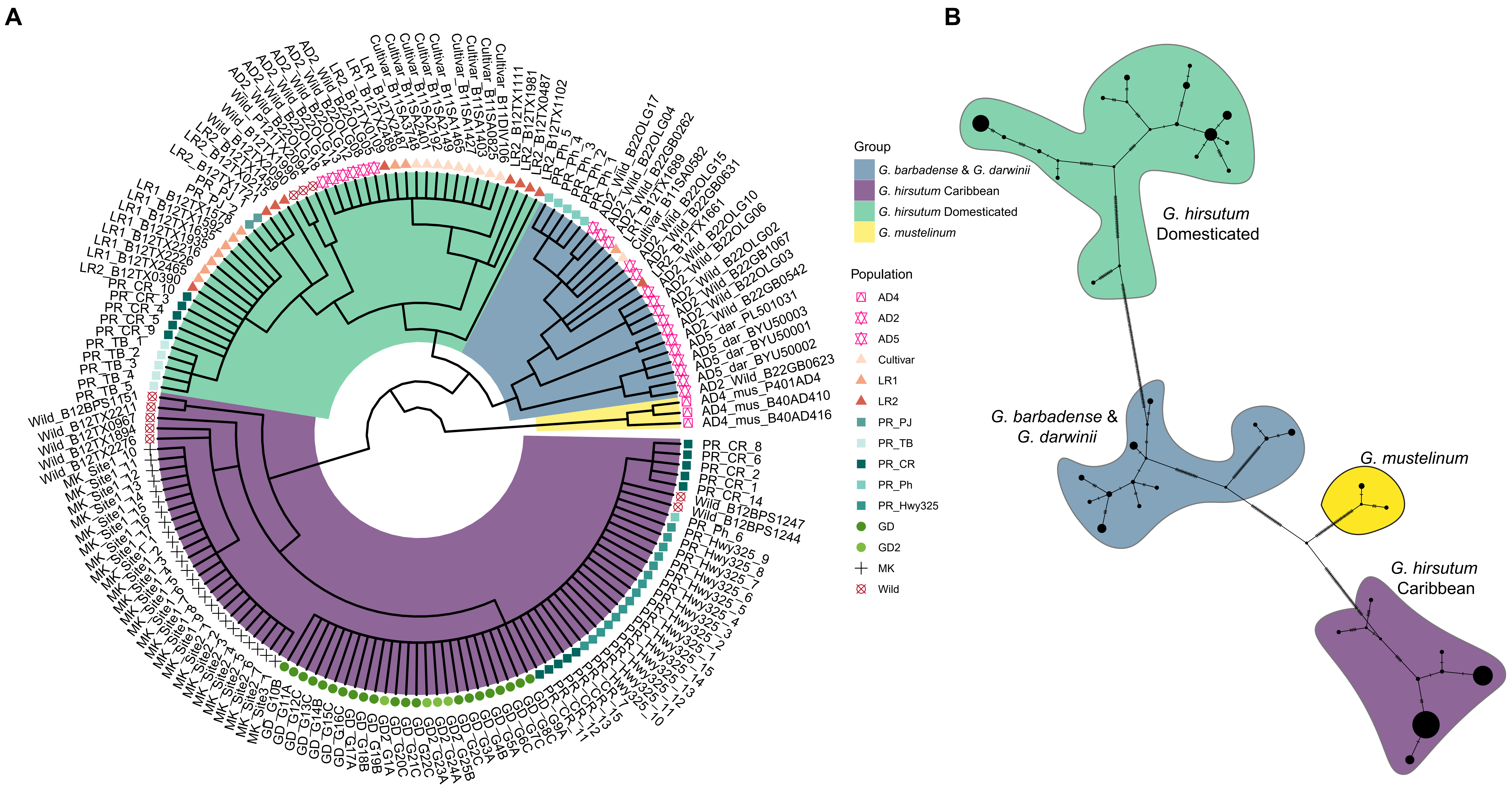
A
B
Group
G. barbadense & G. darwinii
G. hirsutum Caribbean
G. hirsutum Domesticated
G. mustelinum
Population
AD4
AD2
AD5
Cultivar
LR1
LR2
PR_PJ
PR_TB
PR_CR
PR_Ph
PR_Hwy325
GD
GD2
MK
Wild
G. hirsutum Domesticated
G. barbadense & G. darwinii
G. mustelinum
G. hirsutum Caribbean
